# 

**Published:** 1899-11

**Authors:** 


### BOOKS RECEIVED.

The Nervous System and Its Constituent Neurones. Designed for the use of practitioners of medicine and of students of medicine and psychology. By Lewellys F. Barker, M. B., Tor., Associate Professor of Anatomy in the Johns Hopkins University, and Assistant Resident Pathologist to the Johns Hopkins Hospital. With two colored plates and 676 illustrations in the text. Sold by subscription. Cloth, $6.00. New York: D. Appleton & Co. 1899.

Tuttle's Diseases of Children. A manual for students and practitioners. By George M. Tuttle, M. D., Attending Physician to St. Luke's Hospital, Martha

Parsons' Hospital for Children, Bethesda Foundling Asylum, etc., St. Louis, Mo. In one very handsome 12mo volume of 374 pages, with five colored plates. Cloth, $1.50 net. Philadelphia and New York: Lea Brothers & Co.

The International Text-Book of Surgery. By American and British authors. Edited by J. Collins Warren, M. D., LL. D., Professor of Surgery in Harvard Medical School; Surgeon to the Massachusetts General Hospital; and A. Pearce Gould, M. S., F. R. C. S., Surgeon to Middlesex Hospital and Teacher of Operative Surgery at Middlesex Hospital Medical School; Member of the Court of Examiners of the Royal College of Surgeons, England. Volume I. General and Operative Surgery. With 458 illustrations in the text and nine full-page plates in colors. Price, $5.00 net a volume. Philadelphia: W. B. Saunders. 1899.

Transactions of the Texas State Medical Association. Thirty-first annual session, held at San Antonio, April 25, 26, 27 and 28, 1899. Austin: Von Boeckmann, Schutze & Co. 1899.

A Compend of Gynecology. By William H. Wells, M. D., Adjunct Professor of Obstetrics and Diseases of Infancy in the Philadelphia Polyclinic; Instructor of Clinical Obstetrics in the Jefferson Medical College, etc. With illustrations. Philadelphia: P. Blakiston's Son & Co. 1899.

Love and Its Affinities, By George F. Butler, M. D., Professor of Materia Medica and Clinical Medicine in the College of Physicians and Surgeons, Medical Department of the University of Illinois; Author of "Materia, Therapeutics and Pharmacology," etc. Octavo, cloth, gilt top, 134 pages, with photogravure frontispiece, "Cupids Sharpening Their Arrows," by Raphael Mengs. Chicago: G. P. Engelhard & Co., Publishers. 1899.

International Clinics. A Quarterly of Clinical Lectures on Medicine, Neurology, Surgery, Gynecology and Obstetrics, Ophthalmology, Laryngology, Pharyngology, Rhinology, Otology and Dermatology, and Specially Prepared Articles on Treatment and Drugs. By professors and lecturers in the leading medical colleges of the United States, Germany, Austria, France, Great Britain and Canada. Edited by Judson Daland, M. D. (Univ. of Penna.), Philadelphia, Instructor in Clinical Medicine and Lecturer on Physical Diagnosis in the University of Pennsylvania; Assistant Physician to the Hospital of the University of Pennsylvania, etc.; Fellow of the College of Physicians of Philadelphia. Volume III. Ninth series. 1899. Octavo, pp. x.—298. Philadelphia: J. B. Lippincott Co. 1899.

System of Diseases of the Eye. By American, British, Dutch, French, German and Spanish authors. Edited by William F. Norris, A. M., M. D., and Charles A. Oliver, A. M., M. D., of Philadelphia. Volume IV. Motor apparatus, cornea, lens, refraction, medical ophthalmology. Imperial octavo, pp. xii.—962. With fifty-one full-page plates and 211 text illustrations. Philadelphia: J. B. Lippincott Co. 1900.

The Johns Hopkins Hospital Reports. Volume VIII., Nos. 1-2. Contents: On the Rôle of Insects, Arachnids and Myriapods, as Carriers in the Spread of Bacterial and Parasitic Diseases of Men and Animals. A Critical and Historical Study. By George H. F. Nuttall, M. D., Ph. D. Baltimore: The Johns Hopkins Press. 1899.

Malsbary's Practice of Medicine. A Pocket Text-Book of Theory and Practice of Medicine. By George E. Malsbary, M. D., Assistant to the Chair of Theory and Practice of Medicine, Medical College of Ohio, Cincinnati. Just ready. In one handsome 12mo volume of 405 pages, with forty-five illustrations. Cloth, $1.75 net.

A Text-Book of Physiology. For students and practitioners. By Winfield S. Hall, A. M., M. D., Ph. D., Professor of Physiology in the Northwestern University Medical School, Chicago. In one very handsome octavo volume of 672 pages, with 343 engravings and six colored plates. Cloth, $4.00 net; leather, $5.00 net. Philadelphia and New York: Lea Brothers & Co., Publishers.

A Laboratory Manual of Physiological Chemistry. By Elbert W. Rockwood, B. S., M. D., Professor of Chemistry and Toxicology in the University of Iowa. Illustrated with one colored plate and three plates of microscopic preparations. 5⅜ x 7¾ inches. Pages viii.—204. Extra cloth, $1.00 net. Philadelphia: The F. A. Davis Co., Publishers, 1914-16 Cherry Street.

